# *Bacillus subtilis* revives conventional antibiotics against *Staphylococcus aureus* osteomyelitis

**DOI:** 10.1186/s12934-021-01592-5

**Published:** 2021-05-17

**Authors:** Fan Zhang, Bowei Wang, Shiluan Liu, Yuhui Chen, Yihuang Lin, Zixian Liu, Xianrong Zhang, Bin Yu

**Affiliations:** 1 Division of Orthopaedics and Traumatology, Department of Orthopaedics, Nanfang Hospital, Southern Medical University, No.1838 North of Guangzhou Avenue, Guangzhou, 510515 Guangdong China; 2 Guangdong Provincial Key Laboratory of Bone and Cartilage Regenerative Medicine, Nanfang Hospital, Southern Medical University, Guangzhou, China

**Keywords:** *Staphylococcus aureus*, Osteomyelitis, Antibiotic tolerance, *Bacillus subtilis*, Membrane permeabilization, Biofilm

## Abstract

As treatment of *Staphylococcus aureus* (*S. aureus*) osteomyelitis is often hindered by the development of antibiotic tolerance, novel antibacterial therapeutics are required. Here we found that the cell-free supernatant of *Bacillus subtilis* (*B. subtilis* CFS) killed planktonic and biofilm *S. aureus*, and increased *S. aureus* susceptibility to penicillin and gentamicin as well. Further study showed that *B. subtilis* CFS suppressed the expression of the genes involved in adhesive molecules (*Cna* and *ClfA*), virulence factor *Hla*, quorum sensing (*argA*, *argB* and *RNAIII*) and biofilm formation (*Ica* and *sarA*) in *S. aureus*. Additionally, our data showed that *B. subtilis* CFS changed the membrane components and increased membrane permeabilization of *S. aureus*. Finally, we demonstrated that *B. subtilis* CFS increased considerably the susceptibility of *S. aureus* to penicillin and effectively reduced *S. aureus* burdens in a mouse model of implant-associated osteomyelitis. These findings support that *B. subtilis* CFS may be a potential resistance-modifying agent for β-lactam antibiotics against *S. aureus*.

## Introduction

Gram-positive *Staphylococcus aureus* (*S. aureus*) has been identified as the most common causative pathogen for osteomyelitis and other various musculoskeletal infections [1, 2]. *S. aureus* osteomyelitis remains a significant healthcare problem in China and around the world due to high rates of recurrence and treatment failure [3, 4]. Treatment of *S. aureus* infection in bone is complicated by its vast immune evasion, persistence mechanisms and intrinsic antibiotic resistance mechanism. *S. aureus* may secrete multiple virulence factors including immunomodulatory proteins, toxins and superantigens, leading to death of innate immune cells and disturbance of complement activation [5]. As the infection persists and becomes chronic, *S. aureus* may adhere to implanted devices, lacunae-canaliculi in cortical bone or sequestra, thereby forming biofilm phenotype [6, 7]. Once a biofilm forms, *S. aureus* is 10 − 1,000 times more resistant to antimicrobial agents than planktonic bacteria [8] and induces phagocytosis dysfunction of macrophages [9]. Additionally, intracellular persistence of *S. aureus* in osteoblasts, macrophages, osteoclasts or osteocytes may induce immune cell evasion and antibiotic tolerance of *S. aureus* during infection [10, 11]. Furthermore, *S. aureus* has such intrinsic mechanism for antibiotic resistance as decreasing permeability of outer membrane, activating drug efflux systems, and producing excessive β-Lactamase [12–14].

Surgical debridement of necrotic bone combined with long-term administration of antibiotics is a traditional therapy to treat chronic osteomyelitis [15]. Several antibiotics are used for management of *S. aureus* osteomyelitis, such as vancomycin, tobramycin, daptomycin and clindamycin, but the rapid acquisition of resistance to antibiotics by *S. aureus* is a significant problem [16–19]. Therefore, it is urgent to find a more effective antibacterial strategy to prevent occurrence and recurrence of bone infections.

Recently, probiotics such as *Bacillus subtilis* (*B. subtilis*) has been used to prevent infection, because it is a nonpathogenic Gram-positive bacterium which can effectively maintain a beneficial microflora balance in the gastrointestinal tract of a mammalian host [20]. Accumulating evidence from animal and *in vitro* studies suggests that *B. subtilis* produces various substances, such as sufactins, iturins and fengycins, which may benefit anti-bacterial, anti-inflammatory and immunomodulatory applications [21, 22]. Specifically, a recent report showed that the secreted substance from *B. subtilis* abolished colonization with *S. aureus* by suppressing production of the *Arg*-quorum-sensing signaling system [21]. In light of recent evidence implicating anti-infection and decolonization role of *Bacillus* lipopeptides against *S. aureus*, we investigated the effect of *B. subtilis* cell-free supernatant (*B. subtilis* CFS) on the growth of *S. aureus in vitro* and *in vivo.*

Here we found that *B. subtilis* CFS exerted a potent antimicrobial function against *S. aureus* and increased its susceptibility to antibiotics as well *in vitro* and *in vivo* as well. Furthermore, we demonstrated that *B. subtilis* CFS changed the membrane components and increased membrane permeabilization of *S. aureus*, which may be associated with increased susceptibility of *S. aureus* to antibiotics. Our data may suggest a potential application of *B. subtilis* CFS as an adjuvant to potentiate β-lactam antibiotics against *S. aureus* osteomyelitis.

## Materials and methods

### Bacterial strains and culture

*Staphylococcus aureus* strains were isolated from the osteomyelitis subjects from Department of Orthopedics, Nanfang Hospital, Southern Medical University, using PHOENIX 100 (Becton Dickinson Microbiology System, USA). *B. subtilis* (CMCC-B-63,501) was obtained from China General Microbiological Culture Collection Center. Bacterial strains were cultured in TSB (Cat. LA0110, Solarbio, Beijing, China) at 37 ℃ under shaking at 200 rpm. Overnight bacterial cultures were collected by a centrifuge, and pellets washed and resuspended in phosphate-buffered saline (PBS) (Cat. C10010500BT, GIBCO, Beijing, China). The bacterial suspensions were adjusted to an optical density at 600 nm (OD_600_) of 0.5 measured using a microplate spectrophotometer (CLARIOstar, BMG LABTECH, Germany), approximately equal to 1 × 10^8^ colony forming unit per ml (CFU/ml).

### Preparation of cell-free supernatant from *B. subtilis* culture and treatments

To prepare *B. subtilis* CFS, *B. subtilis* strains were cultured at 37℃ under shaking at 200 rpm overnight until the cultures reached an OD_600_ of 0.4 ± 0.05. The CFS of bacterial culture was collected by centrifugation at 6000* g* for 10 min, and then filtered through a 0.22 μm sterilizing-grade filter (Millipore, SLGV033RB, USA) to remove bacteria. The CFS was aliquoted and stored at − 20 ℃ until the day of experimentation.

To evaluate the effect of *B. subtilis* CFS on *S. aureus* genes expression, overnight culture of *S. aureus* strains was collected by a centrifuge, washed with PBS, re-suspended at 1 × 10^8^ CFU/ml in TSB/PBS (1:1 v/v, control) or TSB/*B. subtilis* CFS (1:1 v/v) and incubated in 6-well-plate at 37℃ for 3 h. Finally, bacteria were collected for RNA extraction and analysis of genes expression.

### Planktonic bacterial growth assay

To determine the antibacterial effect of *B. subtilis* CFS on *S. aureus*, the growth of planktonic *S. aureus* was assessed using the method as described previously [23] with some modifications. Briefly, 100 µL of *S. aureus* suspension (5 × 10^8^ CFU/mL) from a fresh overnight culture was inoculated into 5 mL TBS/PBS (1:1 v/v, control) or TSB/*B. subtilis* CFS (1:1 v/v), and incubated with shaking at 200 rpm at 37 ℃. The growth of *S. aureus* was determined by monitoring OD_600_ of the cell culture at 2, 4, 6, 8, 10, 12 and 24 h after seeding.

### Biofilm formation and viability assay of biofilm *S. aureus*

To evaluate the effect of *B. subtilis* CFS on *S. aureus* biofilm formation, 100 µL of *S. aureus* (5 × 10^8^ CFU/mL) was added to 900 µL of TSB/PBS (1:1 v/v), TSB/*B. subtilis* CFS (1:1 v/v), TSB/PBS (1:1 v/v) with 32 µg/mL penicillin, or TSB/PBS (1:1 v/v) with 0.75 µg/mL gentamicin in each well on a 24-well plate and incubated at 37℃ for indicated time points without shaking. Next, after the medium removed, the wells were washed three times with sterile PBS. Finally, the plates were air-dried for 45 min and the adherent cells and matrix were stained with 0.1 % crystal violet solution. To quantify the biofilm production, crystal violet was extracted by incubation in solution (95 % ethanol and 0.1 % acetic acid) at room temperature for 15 min, and absorbance was measured at 600 nm in a microplate reader.

SYTO9 (Cat. S34854, Invitrogen, Thermo Fisher Scientific) and propidium iodide (PI) (Cat. P346, DOJINDO, Japan) staining was performed to evaluate the effect of *B. subtilis* CFS on the viability of biofilm *S. aureus*. 100 µL of *S. aureus* (5 × 10^8^ CFU/mL) was added to 900 µL of TSB in each well on a 12-well plate. After 24 h of static incubation at 37℃, the wells were washed three times with PBS to remove nonadherent cells and refilled with 1 mL/well of the four different sterile culture media: TSB/PBS (1:1 v/v, control), TSB/*B. subtilis* CFS (1:1 v/v), TSB/PBS (1:1 v/v) with 32 µg/mL penicillin, and TSB/PBS (1:1 v/v) with 0.75 µg/mL gentamicin. After 8 h incubation and washing for three times, the biofilm *S. aureus* were stained with 3 µM of PI and 10 µM of SYTO9 in 1 × PBS for 20 min in the dark, and visualized under a fluorescence microscope. Both live and dead bacteria were stained green, and dead ones red.

### Minimum inhibitory concentration (MIC) and killing assay

The potential of synergy was evaluated via MIC evaluation and time-killing assays. MIC was determined using Epsilometer testing (E-test) following the method previously described [24, 25]. Briefly, fresh overnight culture of *S. aureus* was collected and washed twice with PBS, and suspended and pretreated in 1 ml PBS (control) or *B. subtilis* CFS at 1 × 10^8^ CFU/ml for 1 h. 150 µl pretreated *S. aureus* suspension was added and spread evenly on a Mueller-Hinton agar plate. The plate was allowed to dry for 10–15 min before applying E-test strip immobilized with predefined continuous and stable gradients of penicillin (Cat. 921,021, Liofilchem, Italy) or gentamicin (Cat. 920,090, Liofilchem, Italy). The plates were incubated at 35℃ for 24 h and the MIC value was read at the point where the ellipse intersects the E-test strip.

To monitor the response of *B. subtilis* CFS-pretreated *S. aureus* to penicillin or gentamicin, bacterial growth was continuously monitored over a time-course of 24 h (0, 2, 4, 6, 8, 10, 12, 14, 24 h). 500 µl of *S. aureus* suspension (1 × 10^8^ CFU/ml) pretreated with PBS (control) or *B. subtilis* CFS was inoculated into 4.5 mL of Mueller-Hinton broth with penicillin or gentamicin at 0.5 MIC. A 200µL of sample was removed from each tube at indicated time points for measuring OD_600_.

For time-killing assay, 500 µl of *S. aureus* suspension (1 × 10^7^ CFU/ml) pretreated with PBS (control) or *B. subtilis* CFS was inoculated into 4.5 mL of Mueller-Hinton broth with penicillin or gentamicin, with each drug tested at 2 × MIC and 4 × MIC. A 10 µL of sample was removed from each tube at 0, 0.5, 1, 2, 4, 6, 8, 12 and 24 h for colony count enumeration. 10 µL samples with 100-fold dilutions were plated onto Mueller-Hinton agar plates and incubated at 35℃ for 18 h. Colonies were counted and the mean CFU/mL from triplicate samples was evaluated.

### RNA extraction and Quantitative real-time PCR (qRT-PCR)

Total RNA of *S. aureus* was extracted with a Bacterial RNA Extraction Kit (B518655-0050, Sangon Biotech, Shanghai, China) following the manufacturer’s instructions. RNA purity was checked using a NanoDrop spectrophotometer (ND-1000, Nanodrop, USA). RNA was reversely transcribed using the 5× PrimeScript RT Master Mix (RR036A, Takara, Shiga, Japan) according to the manufacturer’s instructions. qRT-PCR was performed using TB Green Premix Ex Taq II (RR820A, Takara, Shiga, Japan). The primers sequences are listed in Table 1. Fold change in level of chosen genes expression were determined using 2^−ΔΔCt^ method with *gyrB* as a housekeeping gene.

**Table 1:** Primers used for quantitative real-time polymerase chain reaction

Genes	Forward primers	Reverse primers
*cna*	5′-AAAGCGTTGCCTAGTGGAGA-3′	5′-AGTGCCTTCCCAAACCTTTT-3′
*clfA*	5′-ATTGGCGTGGCTTCAGTGCT-3′	5′-CGTTTCTTCCGTAGTTGCATTTG-3′
*IcaA*	5′-ACACTTGCTGGCGCAGTCAA-3′	5′-TCTGGAACCAACATCCAACA-3′
*sarA*	5′-TCTTGTTAATGCACAACAACGTAA-3′	5′-TGTTTGCTTCAGTGATTCGTTT-3′
*argA*	5′-GAAGACGATCCAAAACAAAGAG-3′	5′-GTCATTCATATTTTTAGCTTGCTC-3′
*argB*	5′-CCAGTTTGCCACGTATCTTC-3′	5′-GCACCATGTGCATGTCTTC-3′
*RNAIII*	5′-GAAGGAGTGATTTCAATGG-3′	5′-TAAGAAAAATACATAGCACTGA-3′
*hla*	5′-GAAAGGTACCATTGCTGGTCA-3′	5′-AAGGCCAGGCTAAACCACTT-3′
*mecA*	5′-CCTCTGCTCAACAAGTTCCA-3’	5′-ACGTTGTAACCACCCCAAGA-3′
*gyrB*	5′-TTATGGTGCTGGACAGATACA-3′	5′-CACCGTGAAGACCGCCAGATA-3′

### Transmission electron microscopy (TEM)

*Staphylococcus aureus* suspension (1 × 10^8^ CFU) pretreated with PBS (control) or *B. subtilis* CFS was collected and fixed in 2.5 % Glutaric dialdehyde at 4℃ overnight. After washing, *S. aureus* pellets were dehydrated in a series of ethanol concentrations (50–100 %) followed by 100 % acetone. Samples were then embedded in Spurr resin (EM0300, Sigma-Aldrich, USA). 50 nm ultrasections were cut using an ultramicrotome (EM UC7, Leica, Germany) and stained with uranyl acetate for 10 min. After being washed with ddH_2_O, sections were stained with Reynolds lead citrate for 30 min. Finally, sections were observed on a transmission electron microscope (H-7500, Hitachi, Japan) equipped with a 16 million pixels format CCD camera and images were made at 120 kV in high contrast mode.

### Bacterial membrane permeabilization assays

Fresh overnight culture of *S. aureus* (1 × 10^8^ CFU/ml) was treated with PBS or *B. subtilis* CFS for 1 h, then ATP release assay and SYTO9/PI staining were performed to evaluate the changes in membrane permeability of *S. aureus*. SYTO9/PI staining was performed according to the details described in Methods Sect. 2.4. For ATP release assay, the total and extracellular ATP concentrations were detected using BacTiter-Glo™ Microbial Cell Viability Assay Kit (G8230, Promega, USA) and ATP Bioluminescent Assay Kit (FLAA-1KT, Sigma-Aldrich, USA), respectively, according to manufactural instructions. The amount of light produced from samples was measured with the integration time of 6 s in a luminometer (CLARIOstar, BMG LABTECH, Germany). The absorbance values were converted into ATP concentration (nM) based on ATP standard concentration curve.

### Sodium dodecyl sulfate-polyacrylamide gel electrophoresis (SDS-PAGE), immunoblotting and Coomassie brilliant blue (CBB) staining

To detect whether the components of bacterial membrane were affected by *B. subtilis* CFS treatment, proteins from *S. aureus* suspension (1 × 10^8^ CFU/ml, 1 mL) pre-treated with PBS (control) or *B. subtilis* CFS were harvested for analysis with SDS-PAGE. Whole-cell protein (40 µg/lane) and membrane protein (70 µg/lane) were separated with 10 % SDS-PAGE. For CBB G-250 staining, following electrophoresis, the gel was fixed in a solution of 50 % methanol / 10 % glacial acetic acid for 6 h before being stained in the above solution with 0.1 % CBB R-250 for 20 m with gentle agitation. Finally, the light blue background of the gel was eluted with destaining solution (40 % methanol and 10 % glacial acetic acid) before the gel was scanned for further analysis. For immunoblotting, whole-cell protein samples (40 µg/lane) were separated with SDS-PAGE, transferred to PVDF membranes and subjected to immunoblotting analysis. Membranes were probed with antibodies against penicillin-binding protein (PBP)2a (Cat. 130-10307, Raybiotech) and GAPDH (ET1601-4, HUABIO). Proteins were visualized and photographed using Western Lightning Plus ECL (Perkin Elmer) and chemiluminescence instrument (Guangzhou Ewell Bio-Technology Co.Ltd, China). The pixel density of protein bands were analyzed using Image J, the relative level of PBP2a expression was normalized against GAPDH, and fold changes over control were calculated.

### Implant-associated *S. aureus* osteomyelitis mice model

All procedures involving animals were approved by the Animal Care and Use Committee at Nanfang Hospital, Southern Medical University. 88 male C57BL/6 J mice (8–10 weeks old) were obtained from the Animal Center at Southern Medical University. Mice were housed in a facility under specific pathogen-free conditions at 24–27℃ with a 12-h light/dark cycle and had *ad libitum* access to food and water.

The mice model of implant-associated osteomyelitis was made as described previously with modifications [26]. In brief, prior to surgery, they were anesthetized by 125 mg/Kg tribromoethanol (Cat. T831042, Shanghai, China) via intraperitoneal injection. After being shaved and sterilized, an incision was made at the lateral side of the right hind leg and the tibiae was exposed by blunt dissection, and a uni-cortical hole was created at the proximal part of the tibia with a 29-gauge syringe needle. Next, an 8 mm stainless steel pin (0.3 mm in diameter) was inserted into the bone medullary cavity. The hole was sealed with bone wax and the wound was sutured after disinfection. By day 7 post-surgery, *S. aureus* (5 × 10^7^ CFU/mL, 100 µL) was inoculated by intravenous injection via the tail vein. Mice were monitored twice daily for morbidity and mortality.

### Infection and treatments in vivo

To determine the anti-bacterial effect of *B. subtilis* culture CFS *in vivo*, 48 mice with implant-associated *S. aureus* osteomyelitis were randomly divided into two groups and injected intraperitoneally with 200 µL of *B. subtilis* culture CFS or the same volume of PBS (control) every day from the day challenged by *S. aureus*. By days 3 and 14 after *S. aureus* inoculation, the right tibias were collected aseptically and the implanted stainless steel pin was pulled out for analysis of bacterial burden.

To evaluate the responses of *B. subtilis* CFS-pretreated *S. aureus* to penicillin *in vivo*, 40 mice were randomly divided into two groups and infected by *S. aureus* (5 × 10^7^ CFU/mL, 100 µL) pretreated in 1 ml *B. subtilis* CFS or PBS (control) at day 7 after implantation surgery. The next day after *S. aureus* challenge, mice were intraperitoneally injected with penicillin (80 mg/Kg/d). All the mice were sacrificed at days 3 and 14 post-infection by cervical dislocation, the right tibias were collected and the implanted pins were removed from the bone for analysis of bacterial burden.

### Antimicrobial assays in vivo

To assess bacterial burden in bone, the right tibia infected by *S. aureus* was dissected aseptically free from soft tissue, and homogenized in 1 ml of PBS. A 10-fold dilution of the bone homogenate was plated in TSB agar plate. Bacterial colonies were counted and calculated following plate incubation at 37℃ for 18 h. Results of bacterial burden were expressed on a log_10_ scale.

To detect bacterial burden on the implant surface, pins were removed carefully from the tibia after the mice were euthanized. The pins were then sonicated in 1 ml of PBS for 5 min to obtain the biofilm bacteria. Each sample was incubated on TSB agar plates at 37℃. After 24 h incubation, the number of bacterial colonies was counted, calculated and expressed on a log_10_ scale.

The survival rates were recorded within 14 days post-infection on *S. aureus* challenged mice. The infection rates were evaluated based on the mice with infected tibia or implant among surviving mice.

### Histological analysis and immunofluorescence

To evaluate the pathological changes in bone, paraffin-embedded samples were sectioned in 5-µm thickness, deparaffinized with xylene and hydrated by ethanol gradient, followed by hematoxylin and eosin (H&E) staining. Quantitative evaluation of the histopathological changes was performed using Smeltzer’s scoring methods [27]. The parameters included intraosseous acute inflammation (0–4), intraosseous chronic inflammation (0–4), periosteal inflammation (0–4) and bone necrosis (0–4). A score assigned for each sample was the sum of the scores made from the above 4 parameters by two blinded observers independently.

To detect biofilm *S. aureus* on the implant surface, the pins implanted were removed from the tibia gently by day 14 post infection, rinsed 3 times with PBS and fixed in buffered 4 % paraformaldehyde solution for 24 h. The implants were blocked with 3 % BSA for 1 h and incubated with the rabbit polyclonal anti-*S. aureus* antibody (Cat. ab20920, Abcam) at 4℃ overnight. On the next day, sections were incubated with 594-conjugated secondary antibody (Cat. 712-586-153, Jackson ImmunoResearch, West Grove, PA, USA). Slides were mounted with antifade mounting medium with DAPI (Cat. S2110, Solarbio, Solarbio Life Sciences, China), and images were acquired with a fluorescence microscope (BX63, OLYMPUS, Japan).

### Scanning Electron Microscopy (SEM)

Steel pins were removed from the tibias at day 14 after *S. aureus* infection before fixed in 2.5 % Glutaric dialdehyde at 4℃ for 16 h. After being washed and serially dehydrated in a graded series of ethanol solutions, pins were dried in a critical point dryer (HCP-2; Hitachi, Tokyo, Japan) followed by gold plasma coating (E-1010; Hitachi, Tokyo, Japan). Specimens were imaged using a scanning electron microscope (S-3000 N; Hitachi, Tokyo, Japan).

### Statistical analysis

All experiments were performed for at least three times. Since the sample sizes were relatively small and the sample distributions not normally distributed, the nonparametric Mann-Whitney *U* test was applied to compare the differences between the two groups. For comparison of the survival time between the two groups, Gehan-Breslow-Wilcoxon test was used. For assessment of infection rate, Chi-square test was used. *P* < 0.05 was considered statistically significant. All statistical data were analyzed using SPSS 19.0 software.

## Results

### *B. subtilis* CFS suppresses the growth of planktonic and biofilm *S. aureus*

**Fig. 1:**
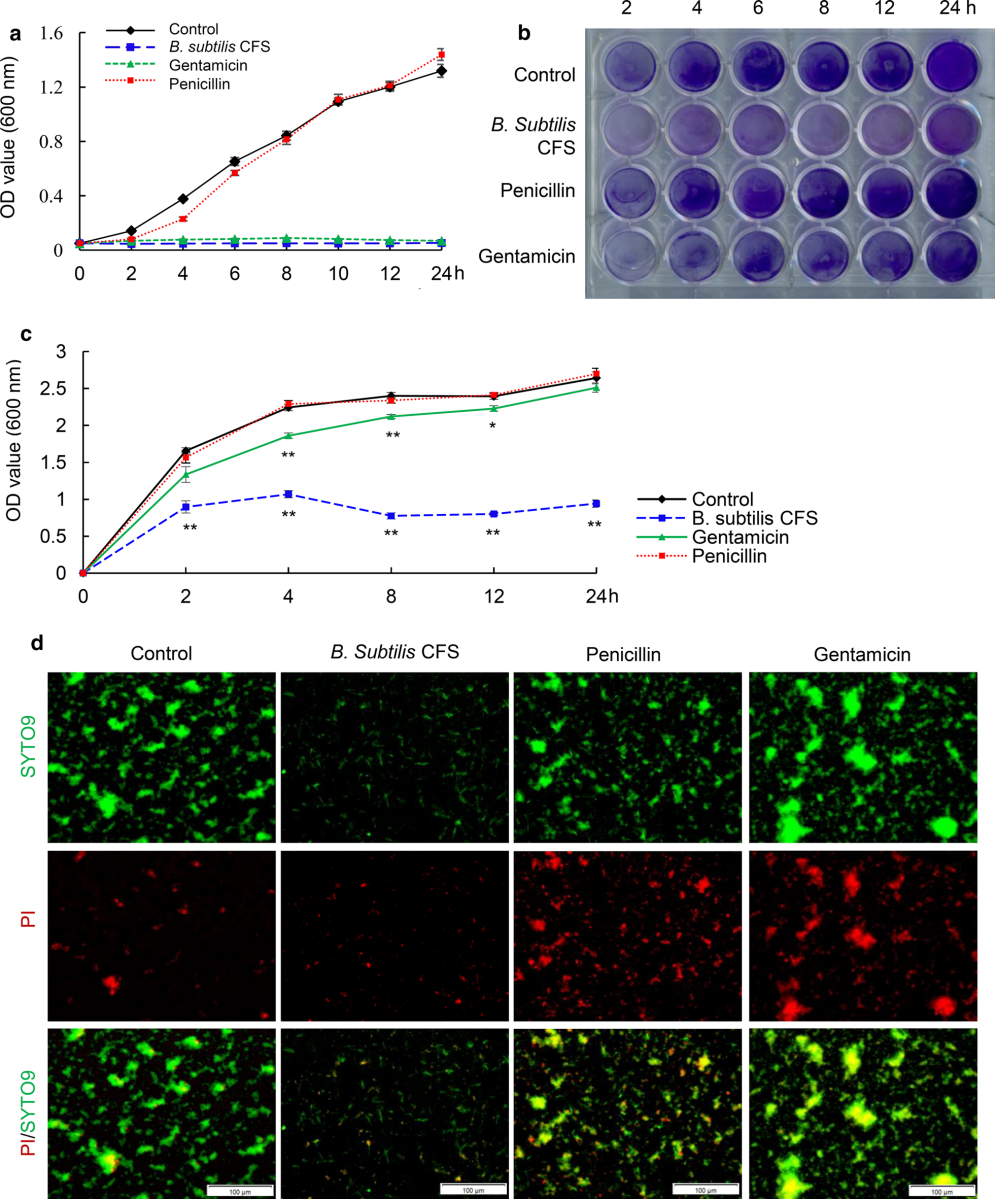
*Bacillus subtilis* cell-free supernatant (*B. subtilis* CFS) inhibits the growth of planktonic and biofilm *Staphylococcus aureus* (*S. aureus*). **a** The growth curve of planktonic *S. aureus*. *S. aureus* (5 × 10^8^ CFU/mL, 100 µL) was grown in TSB/PBS (control), TSB/*B. subtilis* CFS, TSB/PBS with gentamicin (0.75 µg/mL), or TSB/PBS with penicillin (32 µg/mL). Samples were taken out for OD_600_ evaluation at indicated time points. Data are shown as mean ± SE (n = 4 biologically independent samples per time points). **b** Representative images of crystal violet staining for *S. aureus* biofilm. Experiments were repeated independently from 4 different colonies of *S. aureus*. **c** Quantitative analysis of biofilm formation. Crystal violet-staining was dissolved and measured at 600 nm in a microplate reader. N = 4/group, **P < 0.01, Mann-Whitney U test. **d** Representative images of SYTO9-PI staining for biofilm *S. aureus. S. aureus* (5 × 10^7^ CFU/mL) was grown at 37℃ for 24 h, and then treated with TSB/PBS (control), TSB/*B. subtilis* CFS, TSB/PBS with gentamicin (0.75 µg/mL), or TSB/PBS with penicillin (32 µg/mL) for 8 h. After being washing with PBS, biofilm *S. aureus* was examined with SYTO9-PI, followed by analysis using a fluorescence microscope. Both live and dead bacteria were stained green from SYTO9, and dead ones red from PI. Scale bar 100 μm

The investigation of the effect of *B. subtilis* CFS on the growth of *S. aureus* via measuring the OD_600_ of planktonic cells at indicated time points showed that *B. subtilis* CFS significantly suppressed the growth of planktonic *S. aureus* after 4 h of treatment, the inhibitory effect was as strong as that of gentamicin and continued for 24 h of treatment time (Fig. 1a). Moreover, biofilm formation in static *S. aureus* culture was evaluated by crystal violet staining. Results showed much faint staining in the culture of *B. subtilis* CFS-treated *S. aureus* (Fig. 1b), indicating inhibitory effect of *B. subtilis* CFS on *S. aureus* biofilm production. Next, the dissolved crystal violet was subjected to quantitative analysis. As shown in Fig. 1c, B. *subtilis* CFS inhibited biofilm formation during the time points examined, whereas gentamicin had limited inhibitory effect on biofilm production before 12 h of treatment. To evaluate the effect of *B. subtilis* CFS on biofilm *S. aureus*, the *S. aureus* biofilms were formed on plastic wells after static incubation for 24 h, followed by treatment with *B. subtilis* CFS, penicillin or gentamicin for 8 h. Membrane-permeable SYTO9 and membrane-impermeable PI staining was performed to evaluate the amount of biofilm *S. aureus*. Compared to the control group and groups treated by penicillin or gentamicin, *B. subtilis* CFS treatment suppressed both green-stained live biofilm *S. aureus* and red-stained dead ones (Fig. 1d).

### *B. subtilis* CFS increases antibiotic susceptibility of *S. aureus in vitro*

**Fig. 2:**
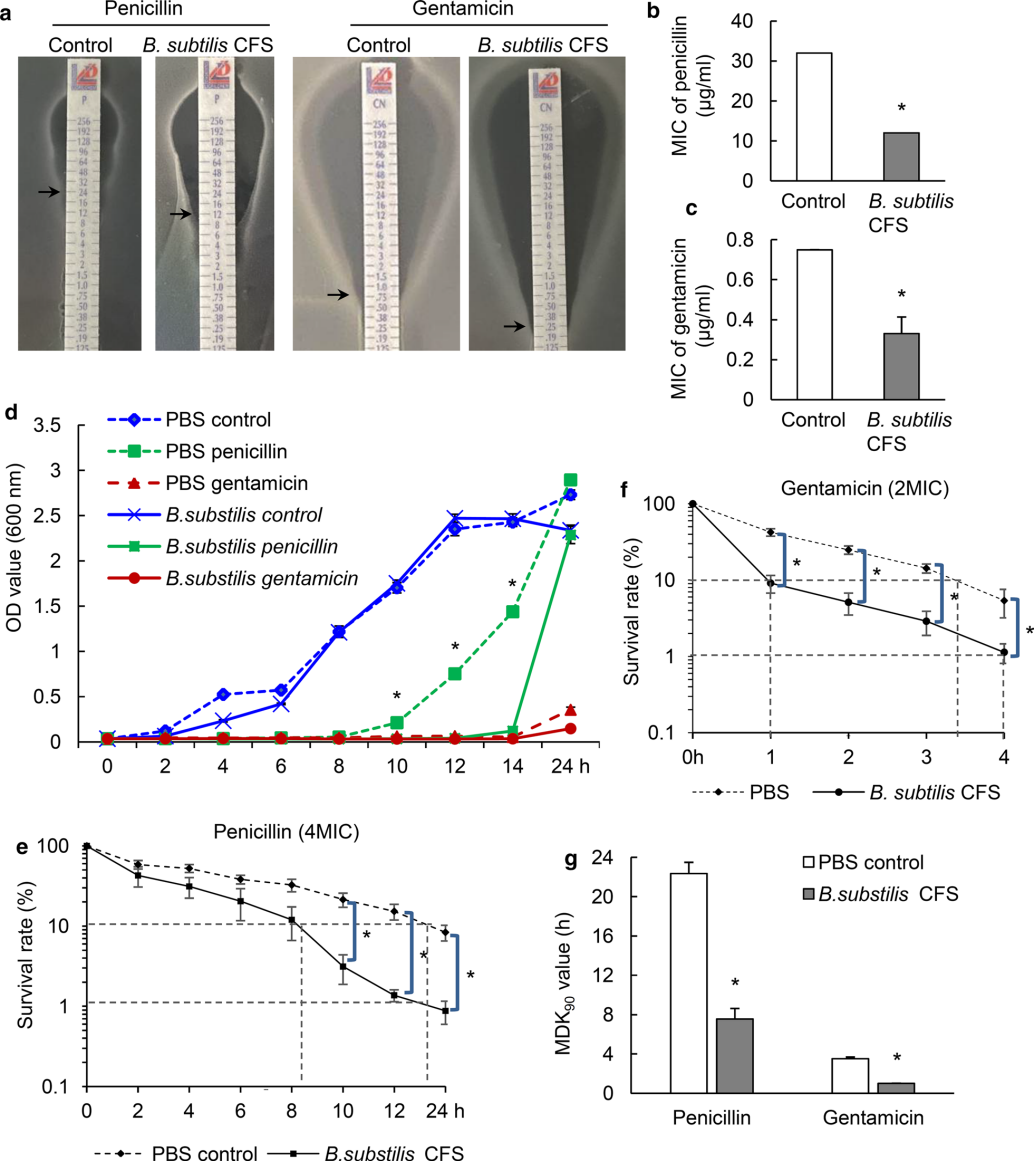
*B. subtilis* CFS increases *S. aureus* susceptibility to penicillin and gentamicin. **a** Representative E-test images of *S. aureus* from penicillin and gentamicin. The minimum inhibitory concentration (MIC) was read off of the strip where the bottom portion of the ellipse intersects with the strip (see black arrows). **b**, **c** Quantitative analysis show significantly decreased MICs of penicillin and gentamicin against *S. aureus* pretreated by *B. subtilis* CFS. MIC values were measured using aliquots of *S. aureus* cultures from three different colonies. *P < 0.05, Mann-Whitney *U* test. **d** The growth of *S. aureus* pretreated with PBS or *B. subtilis* CFS were monitored with or without the presence of penicillin or gentamicin. Fresh overnight culture of *S. aureus* (5 × 10^7^ CFU/mL) was pretreated with PBS or *B. subtilis* CFS for 1 h, and then challenged with PBS, 0.5 MIC penicillin or gentamicin. Samples were collected and OD_600_ was recorded at indicated time points. N = 4/group at each time point. *P < 0.05, Mann-Whitney *U* test. Time-dependent killing of control *S. aureus* and *B. subtilis* CFS-pretreated *S. aureus* by penicillin at 4 × MIC (**e**) and gentamicin at 2 × MIC (**f**). Experiments were independently repeated for 4 times. *P < 0.05, Mann-Whitney *U* test. **g** Minimum duration for killing 90 % (MDK) measurements of control *S. aureus* and *B. subtilis* CFS-pretreated *S. aureus* exposed to penicillin at 4 × MIC or gentamicin at 2 × MIC. Values were determined from the quadruplicate data shown in (shown in **e** and **f**). *P < 0.05, Mann-Whitney *U* test

To evaluate the effect of *B. subtilis* CFS on the response of *S. aureus* to antibiotics, the MIC of *S. aureus* pretreated with PBS or *B. subtilis* CFS for 1 h was detected using E-test, as shown in Fig. 2a. Quantitative analysis showed distinctly decreased MICs of penicillin and gentamicin against *B. subtilis* CFS pretreated-*S. aureus*. Specifically, the MICs of penicillin to PBS-pretreated and *B. subtilis* CFS-pretreated *S. aureus* were 32 µg/ml and 12 µg/ml, respectively (Fig. 2b). The MICs of gentamicin to PBS-pretreated and *B. subtilis* CFS-pretreated *S. aureus* were 0.75 µg/ml and 0.31 ± 0.063 µg/ml, respectively (Fig. 2c). Further study showed that *B. subtilis* CFS pretreatment did not suppress the growth of *S. aureus* in TSB, but increased the susceptibility of *S. aureus* to penicillin. PBS-pretreated *S. aureus* grew rapidly in TSB with penicillin (0.5 MIC) after 8 h of incubation, whereas the growth of *B. subtilis* CFS-pretreated *S. aureus* was substantially suppressed by penicillin after 14 h of incubation (Fig. 2d).

To further examine the effects of *B. subtilis* CFS on antibiotic susceptibility of *S. aureus*, we pretreated *S. aureus* with PBS or *B. subtilis* CFS for 1 h and then performed time-kill assay on planktonic *S. aureus* exposed to penicillin or gentamicin. Since this *S. aureus* strain was not sensitive to penicillin, we therefore made time-kill curves for penicillin at 4× the MIC of antimicrobial concentration. Significantly decreased cell survival rate was observed in *S. aureus* pretreated with *B. subtilis* CFS compared to control ones after 8 h. Additionally, 99 % of *S. aureus* pretreated with *B. subtilis* CFS was killed before 24 h (Fig. 2e). Since this *S. aureus* strain was susceptible to gentamicin, the time-kill curves for gentamicin were made at 2× the MIC of antimicrobial concentration. Results showed that *S. aureus* pretreated with *B. subtilis* CFS had increased sensibility to gentamicin compared to control ones, 99 % of *S. aureus* pretreated with *B. subtilis* CFS was killed before 4 h (Fig. 2f). Based on the above time-kill assay data, the minimum duration for killing 90 % (MDK_90_) values was calculated for *S. aureus* exposed to penicillin or gentamicin. There was a distinct decrease in MDK_90_ values of *S. aureus* pretreated with *B. subtilis* CFS than in those of control ones for both penicillin and gentamicin (Fig. 2g). Together, the above data clearly indicated that pretreatment with *B. subtilis* CFS led to a greater sensitivity of *S. aureus* to penicillin and gentamicin.

### *B. subtilis* CFS increases membrane permeability of *S. aureus*

**Fig. 3:**
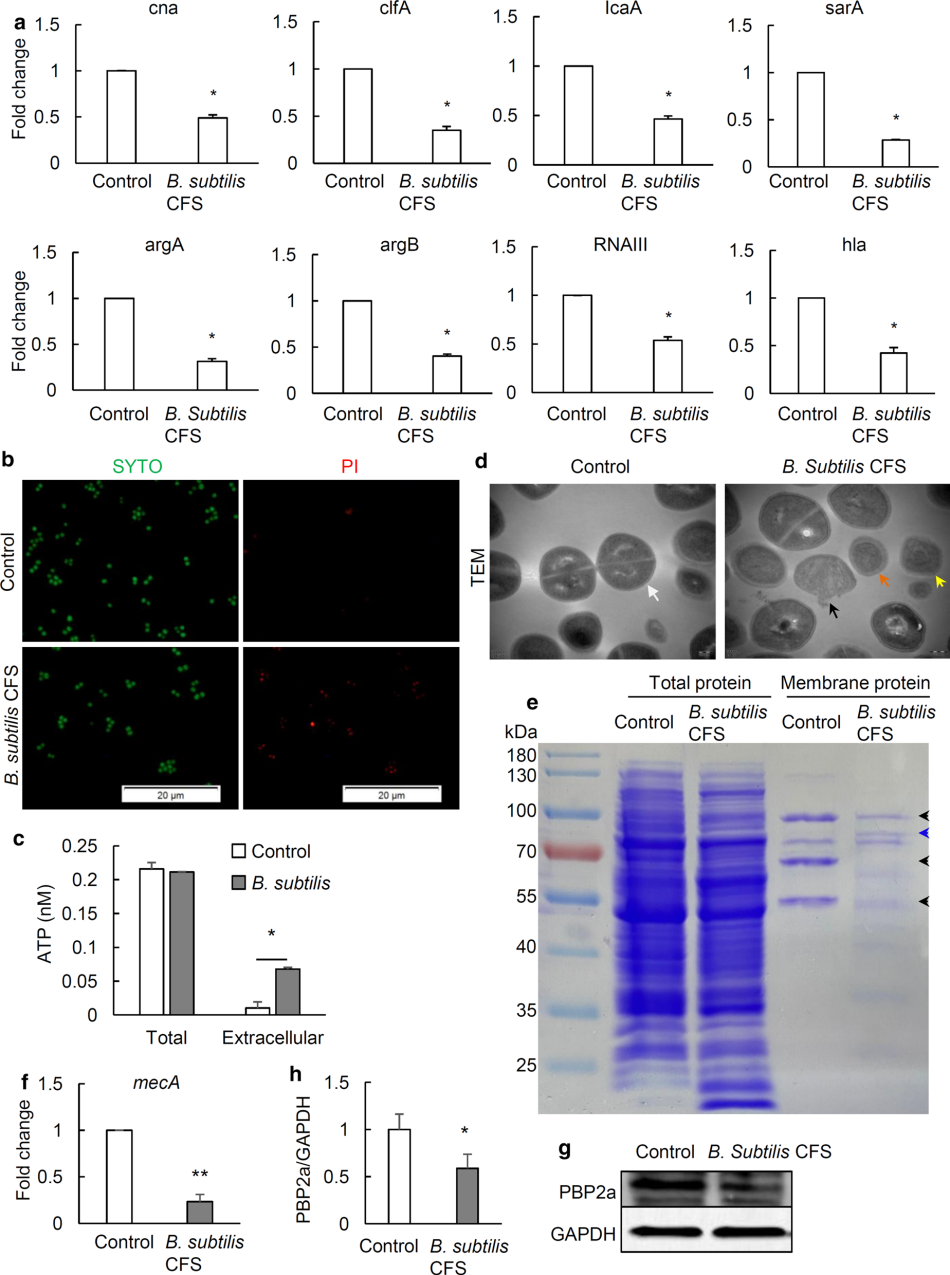
*B. subtilis* CFS alters the pattern of genes expression and increases membrane permeability of *S. aureus*. **a** qRT-PCR analysis of the genes involved in adhesive molecules (*Cna* and *ClfA*), virulence factor *Hla*, quorum sensing (*argA*, *argB* and *RNAIII*) and biofilm formation (*Ica* and sarA) in *S. aureus*. *S. aureus* (1 × 10^8^ CFU/mL) was treated with PBS (control) or *B. subtilis* CFS for 3 h. N = 4/group, *P < 0.05, Mann-Whitney *U* test. **b** Representative images of SYTO9-PI staining to detect the effect of *B. subtilis* CFS on the membrane permeability of *S. aureus*. *S. aureus* (1 × 10^8^ CFU/mL) was treated with PBS (control) or *B. subtilis* CFS for 1 h, followed by staining with 10 µM of SYTO9 (membrane-permeable) and 3 µM of PI (membrane-impermeable). Both live and dead cells were stained with green, and dead ones stained red. Scale bar 20 μm. **c** The leakage of cellular ATP from *S. aureus* after treatment with *B. subtilis* CFS. Data are represented as means ± SD of 4 independent colonies. *P < 0.05 vs. control. Mann-Whitney *U* test. **d** Representative TEM images of PBS-treated (control) and *B. subtilis* CFS-treated *S. aureus*. Scale bar 200 nm. The white, black, yellow, and orange arrows indicate the normal cell, cells with extrusion of intracellular content, the disruption of cell wall and the displacement of cell membrane, respectively. **e** Representative images of Coomassie brilliant blue (CBB) staining for whole-cell and membrane proteins of PBS-treated (control) and *B. subtilis* CFS-treated *S. aureus*. Black arrows indicate decreased levels of proteins at 55, 70 and 100 kDa, and the blue arrow increased level of proteins. Experiments were repeated independently from 4 different colonies of *S. aureus*. **f** qRT-PCR analysis of the mRNA expression of *mecA*, the gene encoding PBP2a. *S. aureus* (1 × 10^8^ CFU/mL) was treated with PBS (control) or *B. subtilis* CFS for 3 h. N = 4/group, **P < 0.01, Mann-Whitney *U* test. Western blot analyses (**g**) and quantification (**h**)　of *B. subtilis* CFS on PBP2a protein expression after 3 h treatment. N = 3/group, *P < 0.05, Mann-Whitney *U* test

Next, we analyzed the effects of *B. subtilis* CFS on expression of *S. aureus* genes encoding adhesive molecules (*Cna* and *ClfA*) and virulence factor *Hla*, and genes involved in quorum sensing (*argA*, *argB* and *RNAIII*) and biofilm formation (*Ica* and *sarA*). Results showed that *B. subtilis* CFS treatment significantly down-regulated the mRNA expression of all the above genes (Fig. 3a).

Since the permeabilizing property of bacterial cell membrane is pivotal to penetration of antibiotics, we analyzed the membrane integrity of *S. aureus* using SYTO9-PI assay. Results showed that *B. subtilis* CFS disrupted the membrane of *S. aureus* after pretreatment for 1 h, as evidenced by the presence of PI molecules in *S. aureus* (Fig. 3b).

The effect of *B. subtilis* CFS on the membrane permeabilization of *S. aureus* was determined by ATP leakage assays. Results showed that *B. subtilis* CFS did not change the whole amount of ATP, but significantly increased the levels of extracellular ATP (0.0676 ± 0.0023 nM) compared to control (0.010 ± 0.0005 nM) (*p* < 0.05) (Fig. 3c), indicating that *S. aureus* membrane was profoundly compromised by *B. subtilis* CFS. Indeed, TEM analysis confirmed that *B. subtilis* CFS disrupted the typical semi-rigid structure of *S. aureus*. As can be seen in Fig. 3d, control *S. aureus* cells showed even cell walls, but *B. subtilis* CFS-treated *S. aureus* showed compromised cell walls, such as disruption of cell wall, displacement of cell membrane and extrusion of intracellular content.

To evaluate the effect of *B. subtilis* CFS on the membrane proteins of *S. aureus*, whole-cell and membrane proteins of *S. aureus* were detected using SDS-PAGE and CBB staining. As seen in Fig. 3e, compared with control, *B. subtilis* CFS treatment considerably changed the pattern of whole-cell protein bands in *S. aureus*. The levels of some proteins decreased while some new proteins appeared. Interestingly, these membrane protein bands with molecular weights of 55, 70 and 100 kDa were much weaker than those of the controls, suggesting that *B. subtilis* CFS has a great effect on the level of proteins in membrane. Next, we evaluated the effect of *B. subtilis* CFS on the mRNA expression of *mecA*, a gene encoding PBP2a which has a molecular weight of around 70 kDa [28]. Results showed that *B. subtilis* CFS substantially suppressed the mRNA expression of PBP2a (Fig. 3f). Analyses of the protein levels of PBP2a in whole-cell lysate of *S. aureus* confirmed the inhibitory effect of *B. subtilis* CFS on PBP2a expression (Fig. 3g, h).

### *B. subtilis* CFS reduces a hematogenous implant-associated infection in mice

**Fig. 4:**
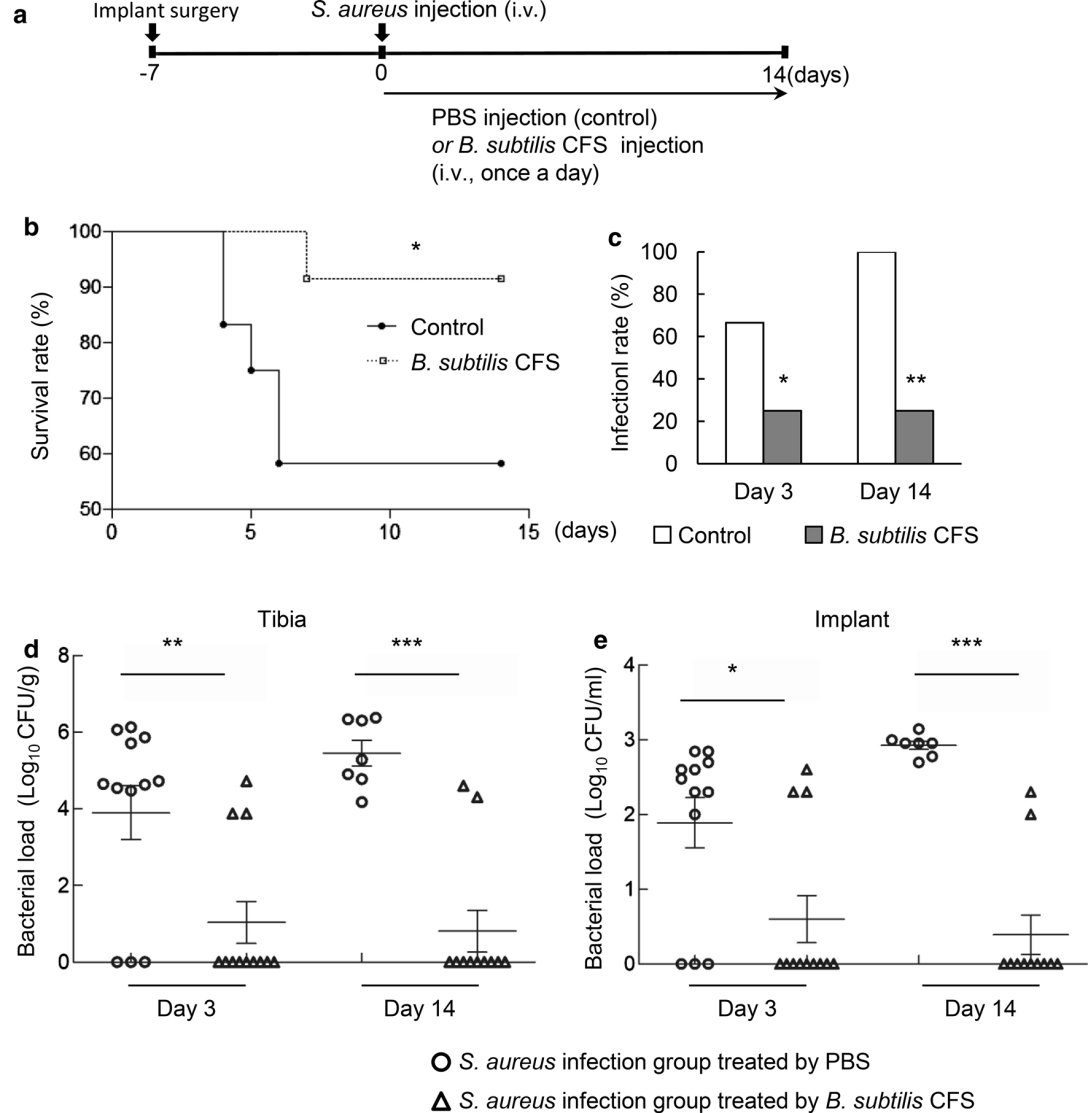
*B. subtilis* CFS suppresses *S. aureus* burden in a mouse model of implant-associated osteomyelitis. **a** Schematic diagram showing establishment of implant-associated *S. aureus* osteomyelitis in mice and treatments. After challenged with *S. aureus*, mice were treated daily with *B. subtilis* CFS or the PBS control. Colony forming unit (CFU) of *S. aureus* was enumerated from the implanted-tibia on days 3 and 14. **b** Survival rate of osteomyelitis mice treated with PBS (control) and *B. subtilis* CFS. Data represent percentage of surviving mice from at least three independent experiments. N = 12/groups, *P < 0.05, Gehan-Breslow-Wilcoxon test. **c** Infection rate in surviving osteomyelitis mice treated with PBS (control) and *B. subtilis* CFS on days 3 and 14 post-infection. N = 12/group, *P < 0.05, **P < 0.01, Chi-square test. **d**, **e** Quantification of *S. aureus* loading recovered from the implanted-tibia (**d**) and the needle (**e**) on days 3 and 14 post-infection. N = 14/group, *P < 0.05, **P < 0.01, ***P < 0.001, Mann-Whitney *U* test

To test whether *B. subtilis* CFS might protect against *S. aureus* infection *in vivo*, we made a mouse osteomyelitis model of hematogenous implant-associated infection. The groups of mice were infected with 5 × 10^6^ CFU of *S. aureus* at day 7 after surgical implantation. Mice were received PBS (control group) or *B. subtilis* CFS injection once a day from the day challenged by *S. aureus* (Fig. 4a). Treatment of *B. subtilis* CFS improved the survival of mice challenged by *S. aureus* compared with control ones (Fig. 4b). The infection rate in surviving control mice increased between days 3 and 14 post-infection. In contrast, surviving mice had a significantly lower infection rate in *B. subtilis* CFS-treated group compared with those in control group, and the infection rate remained unchanged between days 3 and 14 post-infection (Fig. 4c). Accordingly, enumeration of bacterial burdens revealed that control mice harbored higher bacterial burdens on days 3 and 14 post-infection, while *B. subtilis* CFS treatment did substantially reduce bacterial burdens in the tibias and implants (Fig. 4d, e).

**Fig. 5:**
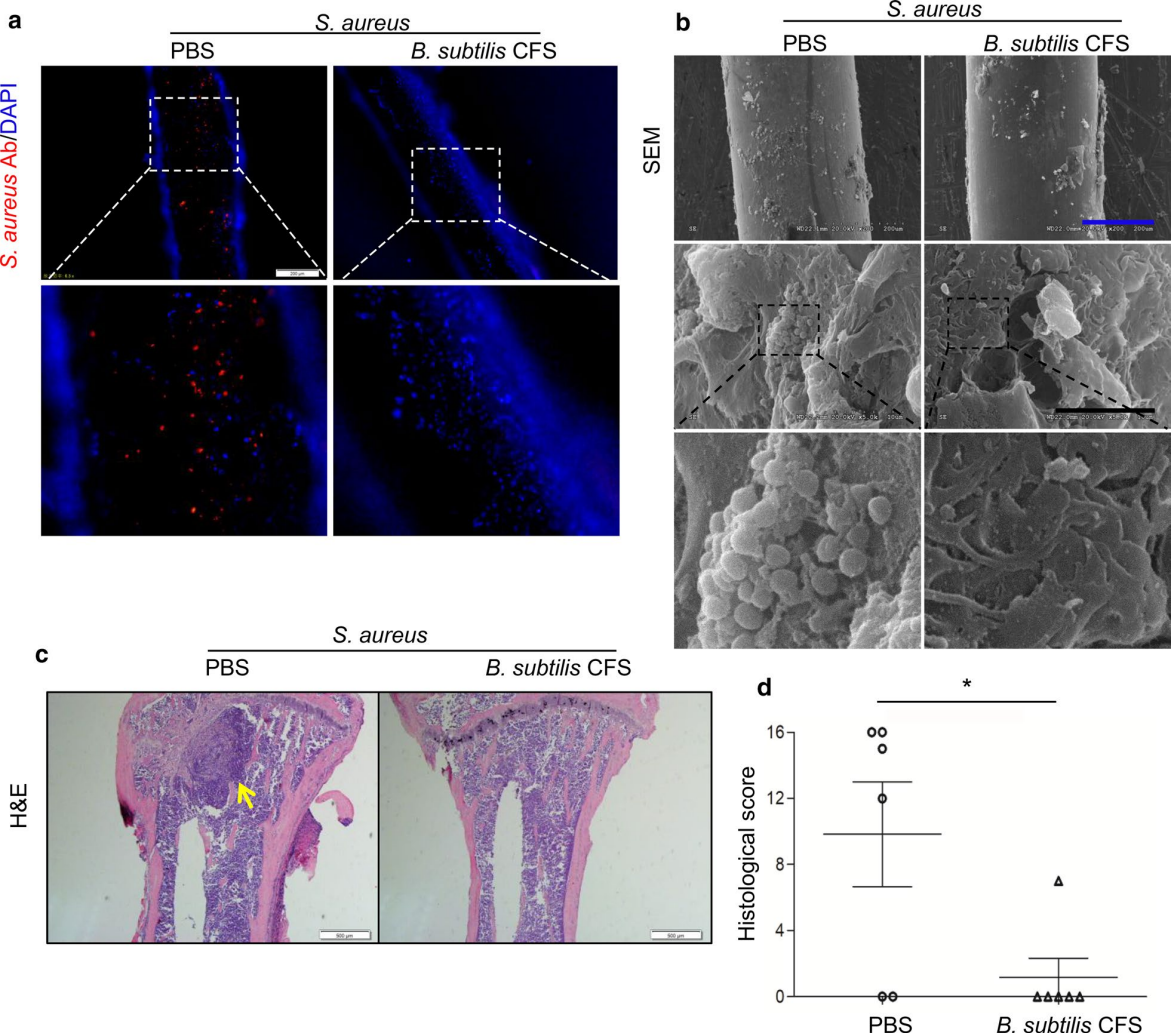
*B. subtilis* CFS suppresses biofilm *S. aureus* in a mouse model of implant-associated osteomyelitis. **a** Representative images of immunofluorescence staining for *S. aureus*. Experiments were repeated independently from 4 samples per group. Scale bar 200 μm. **b** Scanning electron microscopy of *S. aureus* on the implant surface. Experiments were repeated independently from 4 samples per group. Blue scale bar 200 μm and black scale bar 10 μm. **c** Representative images for hematoxylin and eosin (H&E) stained tibial sections from the osteomyelitis mice treated with PBS or *B. subtilis* CFS on day 14 post-infection. Scale bar 500 μm. **d** Histological assessment of H&E stained sections. N = 6/group, *P < 0.05 vs. control. Mann-Whitney *U* test

To detect the effect of *B. subtilis* CFS on growth of biofilm *S. aureus* and changes in bone marrow surrounding an implant, the implants and tibias were harvested on day 14. Immunofluorescence staining showed a considerable amount of *S. aureus*-positive staining on the implant surface in PBS-treated mice, while no obvious signals were observed on the implants in *B. subtilis* CFS-treated mice (Fig. 5a). SEM analysis confirmed biofilm formation rescued by *B. subtilis* CFS treatment (Fig. 5b). Additionally, histologic assessment using H&E staining revealed deformation of bone structure and marked abscess formation within the marrow cavity around the implant in PBS-treated control mice, with no obvious bone destruction in *B. subtilis* CFS-treated mice (Fig. 5c). Histological scores confirmed significantly improved bone structure in the bone of *B. subtilis* CFS-treated mice (Fig. 5d).

### *Staphylococcus aureus* pretreated by *B. subtilis* CFS is susceptible to penicillin *in vivo*

**Fig. 6:**
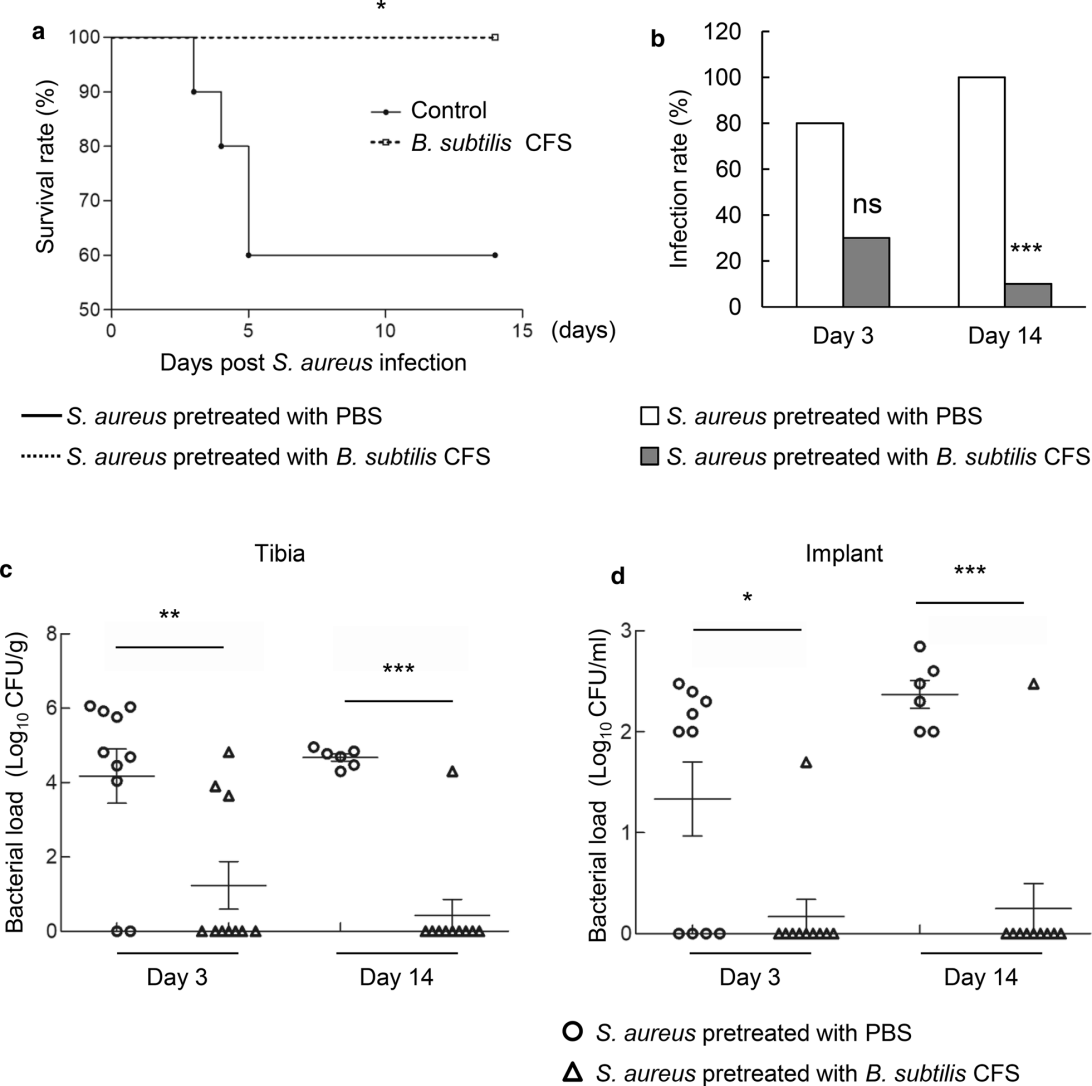
*Bacillus subtilis* cell-free supernatant (*B. subtilis* CFS) increases the susceptibility of *S. aureus* to penicillin. **a** Survival percentage of implant-associated osteomyelitis mice infected by *S. aureus* pretreated with PBS (control) and *B. subtilis* CFS. All mice were treated with penicillin (80 mg/Kg/d) from the day challenged by *S. aureus*. N = 10/groups, *P < 0.05, Gehan-Breslow-Wilcoxon test. **b** Infection rate in surviving mice infected by *S. aureus* pretreated with PBS (control) and *B. subtilis* CFS on days 3 and 14 post-infection. N = 10/group, *P < 0.05, ns *P* > 0.05, Chi-square test. Quantification of *S. aureus* loading recovered from the implanted-tibia (**c**) and the needle (**d**) on days 3 and 14 post-infection. N = 10/group, **P < 0.01, ***P < 0.001, Mann-Whitney *U* test

To address the effect of *B. subtilis* CFS pretreatment on the susceptibility of *S. aureus* to penicillin *in vivo*, we examined the outcomes of penicillin treatment of mice infected by PBS-pretreated or *B. subtilis* CFS-pretreated *S. aureus*. Penicillin treatment did not extend the survival of mice infected by PBS-pretreated *S. aureus* but significantly prolonged the survival of mice infected by *B. subtilis* CFS-pretreated *S. aureus* (Fig. 6a). In surviving mice, penicillin significantly suppressed the infection rate in mice infected by *B. subtilis* CFS-pretreated *S. aureus* (Fig. 6b). Furthermore, enumeration of *S. aureus* cells in the tibias and implants by days 3 and 14 post-infection showed that the surviving mice infected by *B. subtilis* CFS-pretreated *S. aureus* had significantly decreased bacterial burdens in the infected tibias and implants (Fig. 6c, d). Together, these data collected *in vivo* supported an increased susceptibility of *S. aureus* pretreated by *B. subtilis* CFS to penicillin.

## Discussion

*S. aureus* is one of the important pathogens causing various infections like osteomyelitis. It is hard to cure, in part because of the ability of *S. aureus* to enter into an antibiotic-tolerance state and the formation of biofilm *S. aureus*. The present study provided evidence for bactericidal effect of *B. subtilis* CFS on both planktonic and biofilm *S. aureus in vitro* and *in vivo*. We also demonstrated that *B. subtilis* CFS treatment increased the susceptibility of *S. aureus* to penicillin and gentamicin, which might have been associated with changes in membrane components and increased membrane permeability in *S. aureus*, respectively. Furthermore, our findings also demonstrated the sensitivity of *B. subtilis* CFS-pretreated *S. aureus* to penicillin in a mouse model of implant-associated osteomyelitis.

Several studies have reported that *B. subtilis* exerts an antimicrobial effect against a broad spectrum of pathogens through direct bactericidal activity or indirect enhancement of immune response, such as interrupting quorum-sensing regulatory system by production of fengycins [21], inhibiting *S. aureus* adhesion and biofilm formation by production of surfactins [29], and enhancing anti-microbial function of macrophage [30]. In agreement with the above reports, our study has confirmed a potent inhibitory capacity of *B. subtilis* CFS against both planktonic and biofilm *S. aureus in vitro*, which may prominently suppress expression of genes associated with *S. aureus* adhesion, biofilm formation, quorum-sensing and virulence. Furthermore, our data demonstrate the bactericidal effect of *B. subtilis* CFS on biofilm *S. aureus* in a mouse model of implant-associated osteomyelitis.

A critical finding in this study is that *B. subtilis* CFS increased the susceptibility of *S. aureus* to penicillin *in vitro* and *in vivo*. Generally, *S. aureus* strains are found to be resistant to almost all β-lactam antibiotics as they produce β-Lactamase that breaks down β-lactam ring or a penicillin-binding protein called PBP2a that has a low binding affinity to β-lactam antibiotics [14, 31]. Our data demonstrates the inhibitory effect of *B. subtilis* CFS on the expression of PBP2a at both transcriptional and translational level, therefore, the increased sensitivity of *S. aureus* to penicillin may be mainly due to the suppressed level of PBP2a by *B. subtilis* CFS treatment. Additionally, due to the increased membrane permeability of *S. aureus* as detected by SYTO 9/PI staining and ATP leakage assay, *B. subtilis* CFS may also sensitize *S. aureus* to gentamicin, an antibiotic that inhibits protein synthesis.

Increasing evidence has pointed to the importance of functional membrane microdomains in the combat against antibiotic resistance in *S. aureus* and perturbation of functional membrane microdomains assembly may disable bacterial antibiotic resistance [13, 32]. The antimicrobial drugs approved generally target only a fraction of proteins that are involved in membrane or cell wall synthesis [33, 34]. In the present study, *B. subtilis* CFS treatment has been shown to suppress the expression of a bunch of membrane proteins, indicating possible destruction of functional membrane domains in *S. aureus*. Our TEM data supports this mechanism that *B. subtilis* CFS treatment may induce the disruption of cell wall in *S. aureus*.

## Conclusions

*S. aureus* osteomyelitis is difficult to treat, in part because of the increase in prevalence of antibiotic resistant strains of *S. aureus*. Our results shows that *B. subtilis* potentiates the efficacy of conventional antibiotics against *S. aureus.* Although the key components of *B. subtilis* CFS that play an antimicrobial role and the precise mechanism by which *B. subtilis* CFS increases *S. aureus* susceptibility to penicillin require further experimentation, our data strongly suggest that *B. subtilis* CFS may be a promising candidate for novel anti-infective strategies.

## Abbreviations

*S. aureus*: *Staphylococcus aureus*
*B. subtilis*: *Bacillus subtilis*
*B. subtilis* CFS: *B. subtilis* Cell-free supernatant
TSB: Tryptic soy broth
PBS: Phosphate-buffered saline
OD: Optical density
CFU: Colony forming unit
PI: Propidium iodide
MIC: Minimum inhibitory concentration
MDK_90_: Minimum duration for killing 90%
qRT-PCR: Quantitative real-time PCR
PBP2a: Penicillin-binding protein 2a
TEM: Transmission Electron Microscropy
ATP: Adenosine 5’-triphosphate
SDS-PAGE: Sodium dodecyl sulfate–polyacrylamide gel electrophoresis
CBB: Coomassie brilliant blue
H&E: Hematoxylin and eosin
SEM: Scanning electron microscopy
ANOVA: Analysis of variance
